# Structure of ATP synthase from ESKAPE pathogen *Acinetobacter baumannii*

**DOI:** 10.1126/sciadv.abl5966

**Published:** 2022-02-16

**Authors:** Julius K. Demmer, Ben P. Phillips, O. Lisa Uhrig, Alain Filloux, Luke P. Allsopp, Maike Bublitz, Thomas Meier

**Affiliations:** 1 Department of Life Sciences, Imperial College London, Exhibition Road, London SW7 2AZ, UK.; 2 MRC Centre for Molecular Bacteriology and Infection, Department of Life Sciences, Imperial College London, London SW7 2AZ, UK.; 3 National Heart and Lung Institute, Imperial College, London, UK.; 4 Department of Biochemistry, University of Oxford, South Parks Road, Oxford OX1 3QU, UK.; 5 Private University in the Principality of Liechtenstein, Triesen, Liechtenstein.

## Abstract

The global spread of multidrug-resistant *Acinetobacter baumannii* infections urgently calls for the identification of novel drug targets. We solved the electron cryo-microscopy structure of the F_1_F_o_–adenosine 5′-triphosphate (ATP) synthase from *A. baumannii* in three distinct conformational states. The nucleotide-converting F_1_ subcomplex reveals a specific self-inhibition mechanism, which supports a unidirectional ratchet mechanism to avoid wasteful ATP consumption. In the membrane-embedded F_o_ complex, the structure shows unique structural adaptations along both the entry and exit pathways of the proton-conducting a-subunit. These features, absent in mitochondrial ATP synthases, represent attractive targets for the development of next-generation therapeutics that can act directly at the culmination of bioenergetics in this clinically relevant pathogen.

## Teaser

Structure of the ATP synthase from the human pathogen *Acinetobacter baumannii* provides a new target for drug development.

## INTRODUCTION

*Acinetobacter baumannii* is an ESKAPE (*Enterococcus faecium*, *Staphylococcus aureus*, *Klebsiella pneumoniae*, *Acinetobacter baumannii*, *Pseudomonas aeruginosa*, and *Enterobacter* spp.) pathogen (*1*) that causes a variety of hospital-acquired infections and severe treatment complications, including severe acute respiratory syndrome coronavirus 2 (SARS-CoV-2)–associated pneumonia (*2*). A marked increase in multidrug resistance (MDR) in the past decade has placed *A. baumannii* at the top of the World Health Organization’s (WHO’s) priority pathogen list, urgently requiring the development of new drugs with alternative cellular targets (*3*). Recent successes in treating MDR bacteria have included novel inhibitors of the F_1_F_o_-ATP synthase, with bedaquiline (BDQ) having been approved in 2012 as the only novel last-line antibiotic for MDR *Mycobacterium tuberculosis* since rifampicin in 1971 (*4*). Adenosine 5′-triphosphate (ATP) synthase is highly up-regulated in MDR patient strains of *A. baumannii*, and unlike many traditional targets, the enzyme is conditionally essential for survival in biofilms and dormant states (*5*–*8*).

F_1_F_o_-ATP synthases are large, membrane-embedded macromolecular complexes that harness energy from the proton-motive force (pmf) and use it to synthesize ATP via a unique rotary mechanism. The simplest forms of ATP synthase are found in bacteria and plant chloroplasts and have an F_1_ head containing subunits α_3_β_3_γδε and an F_o_ motor containing subunits ab_2_c_9-15_. Protons travel through the membrane-embedded a-subunit turning the c-ring. The rotating c-ring transfers torque to a central shaft comprising the ε- and γ-subunits, which protrude into the α_3_β_3_ hexamer, inducing conformational changes in the F_1_ nucleotide binding pockets, thus converting the rotary force into ATP synthesis. To identify unique vulnerabilities of the *A. baumannii* ATP synthase, we have determined the structure by electron cryo-microscopy (cryo-EM) single-particle analysis and analyzed its structure and mechanism.

## RESULTS AND DISCUSSION

### Purification and structure determination of *A. baumannii* ATP synthase

The *A. baumannii* ATP synthase complex was purified via affinity tag and reconstituted into peptidiscs (fig. S1A). Adenosine triphosphatase (ATPase) hydrolytic activity assays indicated that the complex was in an “autoinhibited state” (*9*) (fig. S1B). The sample was analyzed by single-particle cryo-EM (fig. S1C); 11,490 movies yielded 349,160 particles that were refined into three separate states, distinguished by the position of the central stalk and reached overall resolutions of 3.1, 4.6, and 4.3 Å, respectively (figs. S2 and S3). Further masked refinements improved the initially poor local resolution in the F_o_ region (4 to 7 Å; fig. S3A) of state 1 to 3.7 Å (fig. S3C) and permitted de novo building of the complex into a composite map (fig. S3D). State 1 was then used as a reference to build structures of the remaining two states.

**Fig. 1.**
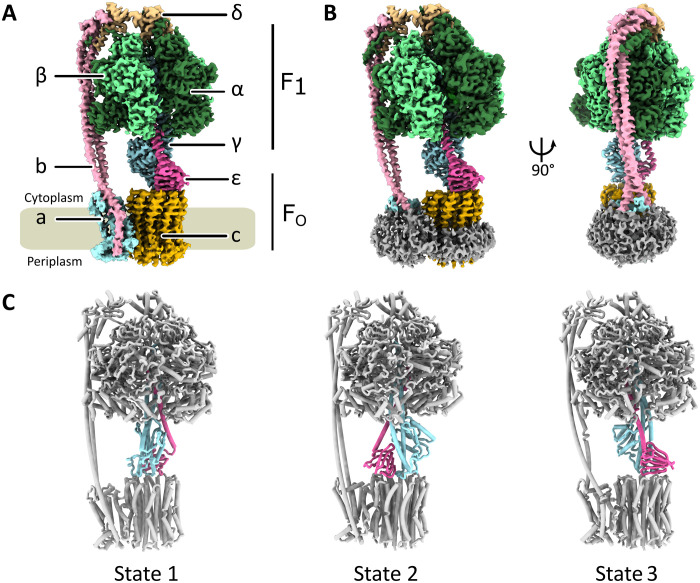
Structure of the F_1_F_o_-ATP synthase from *A. baumannii.* (**A**) Cryo-EM map of *A. baumannii* ATP synthase colored by subunits as indicated. The membrane is indicated in gray. The density representing the peptidisc has been manually removed for increased clarity. (**B**) Views of the state 1 cryo-EM map including peptidisc density shown in gray. (**C**) Cartoon representation of the three rotational states observed for *A. baumannii* ATP synthase, differing in the relative positions of the central stalk shown in light blue (γ) and pink (ε) and the conformations of the α- and β-subunits. Structural alignments of the γ-subunit between states revealed that the three states differ by almost exactly 120°.

The *A. baumannii* ATP synthase has a subunit stoichiometry of α_3_β_3_γδεab_2_c_10_; its overall architecture resembles that of other bacterial and chloroplast ATP synthases (Fig. 1, A to C) (*10*–*12*). The central stalk is rotated by almost exactly 120^o^ between each of the three conformational states (Fig. 1D), as seen previously (*10*), suggesting common stable low-energy intermediate states in bacterial ATP synthases.

### F_1_ complex

**Fig. 2.**
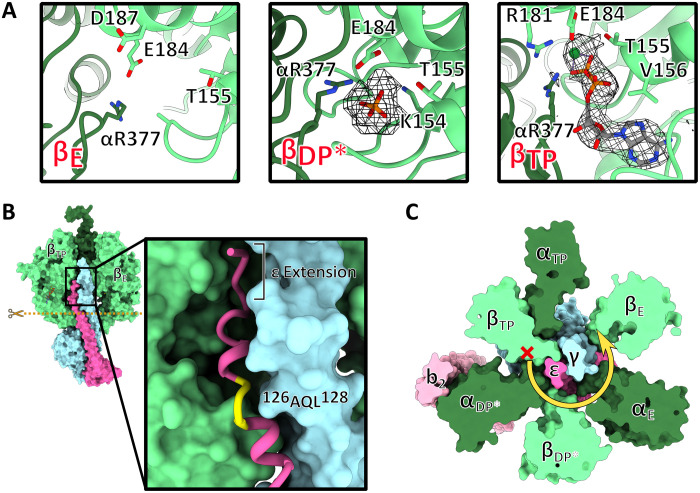
Structural details in the F_1_ complex. (**A**) Close-up views of the catalytic nucleotide binding sites in the β-subunits in cartoon representation. The maps for the nucleotide and phosphate are shown in mesh. (**B**) Structure of the F_1_ complex in surface representation including the central stalk; subunits α_DP_, β_DP*_, and α_E_ removed to reveal how subunits γ and ε insert into the head. Inset: Close-up of the C-terminal extension of ε-subunit inserting between β_TP_ and α_DP*_ (not shown) showing the unwound turn of ε ^126^AQL^128^ (yellow) and the *A. baumannii–*specific C-terminal extension of ε. (**C**) Horizontal section of F_1_ as indicated by the dashed line in (B) reveals mechanism of unidirectional inhibition. Steric clashes between ε and β_TP_ prevent rotation of the central stalk in the ATP hydrolysis direction (red cross) but permit unlocking and rotation in the ATP synthesis direction (yellow arrow).

In F_1_, we observe the canonical “Walker-Boyer” state geometries in both the “open” conformation (β_E_, “empty”) and the “loose” conformation (β_TP_, with bound Mg–adenosine 5′-diphosphate) (Fig. 2A) (*13*, *14*). As in *Bacillus* PS3 (*10*), the third β-subunit (“tight” or β_DP_) is in a “forced open” conformation (denoted as β_DP*_), and we find only weak map features in this region, which we interpret as a loosely bound inorganic phosphate ion (Fig. 2A). This conformation is induced by an interaction between the ε-subunit and a predominantly hydrophobic loop-helix region in the β_DP*_-subunit (^377^DIIAILGMDE^386^), which overlaps with the highly conserved ^385^DELAEED^391^ motif (fig. S4). The forced open conformation of the β_DP*_ site is more similar to the open β_E_ conformation [all-atom root mean square deviation (RMSD) of 1.5 Å] than to the loose β_TP_ (all-atom RMSD β_DP_ to β_TP_ 2.6 Å) (fig. S4) but is less open than the equivalent site in PS3 (all-atom RMSD *A. baumannii* β_DP*_ to *Bacillus* PS3 β_E_ = 0.65 Å).

### Blockage of ATP hydrolysis and F_1_ ratchet mechanism

The *A. baumannii* ε-subunit is similar to those seen in other bacteria, containing an extended C-terminal α helix. However, where PS3 ε terminates, both *A. baumannii* and *Escherichia coli* unwind in a short ^126^AQL^128^ motif (Fig. 2B) (*9*, *10*). While the *E. coli* subunit ε bends here and protrudes “horizontally” between the α_DP_- and β_TP_-subunits, *A. baumannii* ε continues further upward into the F_1_ head, forming two more helical turns followed by a five-residue extension (Fig. 2B), which forms several additional interactions with the γ-, β_TP_-, and α_DP_-subunits. This may further stabilize subunit ε in the inhibitory “up” position, which blocks rotation in the hydrolysis direction, while still enabling ATP synthesis (Fig. 2C) (*10*). The importance of the bacterial ε-subunit C-terminal region has been highlighted in a biochemical study where an *E. coli* ATP synthase mutant with a truncated C terminus showed reduced growth, inhibited ATP synthesis activity, and a markedly reduced capacity to inhibit ATP hydrolysis (*15*). The “ratchet” mechanism seen in *A. baumannii* ATP synthase is similar to that observed in PS3 but distinct from that in the mycobacterial ATP synthase, which instead relies on the formation of a temporary β strand interaction between α- and ε-subunits (*16*). The stronger blockage may help avoid wasteful ATP hydrolysis, enhancing in vivo host persistence.

### F_o_ complex and unique structural features

**Fig. 3.**
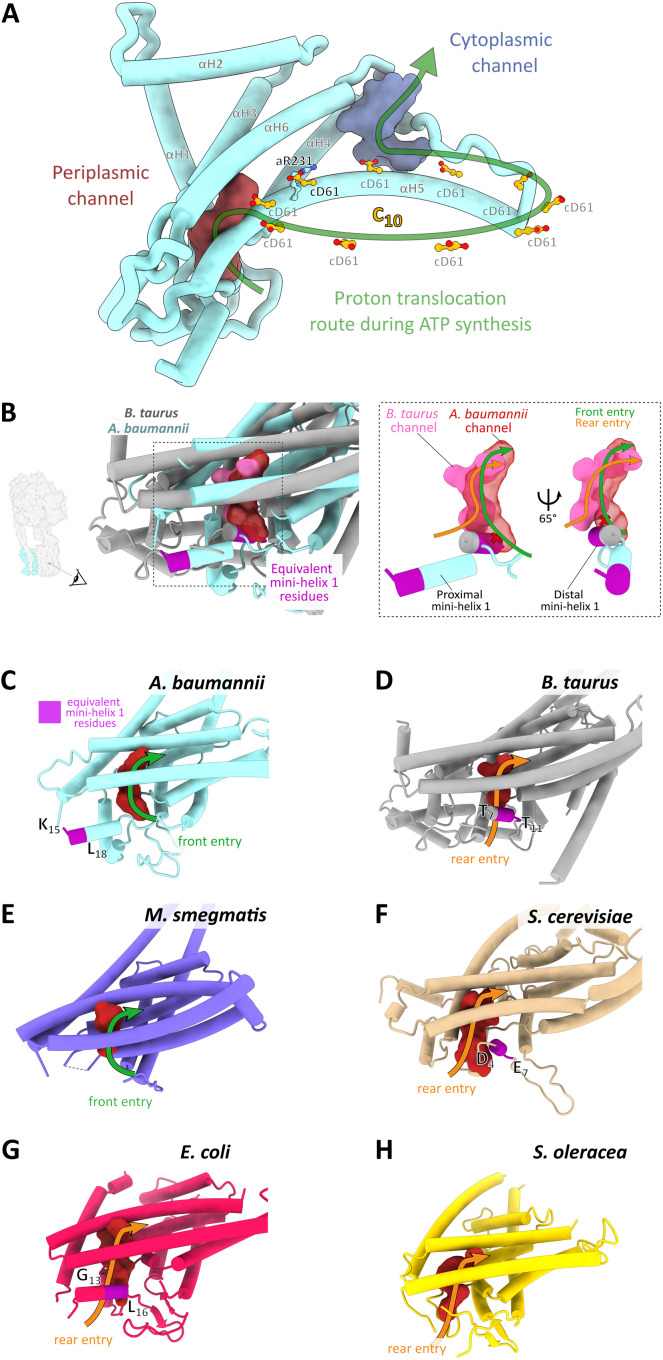
Structure of the F_o_ motor and an *Acinetobacter*-specific proton entry channel site. (**A**) Ribbon representation of the a-subunit with the key conserved aR231 and the protonatable cD61 carboxylates in the c_10_ ring shown in ball-and-stick representation. Periplasmic and cytoplasmic channels are shown in maroon and purple, respectively. Arrow indicates direction of rotation during ATP synthesis. (**B**) Structurally distinct proton entry route into the periplasmic entry channel of *A. baumannii* ATP synthase. A structural alignment of *A. baumannii* (light blue) and *Bos taurus* (gray) a-subunit is shown in cartoon representation, along with the periplasmic entry channels calculated using HOLLOW (*33*). Red channel: *A. baumannii*. Pink channel: *B. taurus*. The dashed rectangle indicates the zoomed region shown in the right panel figure, with a simplified view of the channels shown in two rotations. Viewing direction is indicated in the left panel and is consistent in the remaining panels (B to H). The equivalent mini-helix 1 residues are colored in magenta and correspond to the region indicated in the a-subunit amino acid alignment (fig. S5A). This helix can assume a proximal (*A. baumannii*) or distal (*B. taurus*) position, as indicated. The corresponding proton entry paths from either the front (*A. baumannii*) or from the rear (*B. taurus*) are indicated by green and orange arrows. (**C** to **H**) Same view as in (B) of the a-subunit, the periplasmic channel, the proton entry route, and the position of mini-helix 1 in various species. (C) *A. baumannii*, state 1 (7P2Y); (D) *B. taurus*, 6ZQM (*37*); (E) *M. smegmatis*, 7JGA (*16*); (F) *Saccharomyces cerevisiae*, 6B8H (*38*); (G) *E. coli*, 6OQR (*39*); and (H) *Spinacia oleracea*, 6FKF (*12*). Note that the residues corresponding to mini-helix 1 are not modeled in the *M. smegmatis* and *S. oleracea* structures.

The γε complex is linked to the F_o_ motor, which comprises 10 copies of the c-subunit (c_10_ ring) and one copy of the proton-conducting a-subunit. Its core fold is highly conserved and resembles other ATP synthase structures, while the proton-conducting channels are most similar to those in other bacterial a-subunits (Fig. 3A and fig. S5B). Bacterial ATP synthases contain sequence insertions in the N terminus of the a-subunit, and *A. baumannii* has the largest insertion of the structurally characterized ATP synthases (fig. S5A), resulting in a repositioning of the first short α helix toward the periplasm (Fig. 3, B and C). This shift relocates the entry site of the periplasmic proton channel: In the mammalian ATP, synthase protons enter from behind this mini-helix (Fig. 3E), whereas in *A. baumannii*, they approach from the front, as viewed from the c-ring (Fig. 3, B and C). Positioning of this mini-helix also appears important in determining the channel entry point in other ATP synthases (Fig. 3, C to H). As several inhibitors of ATP synthases bind to the proton entry channel, including organotin compounds currently used as pesticides (*17*, *18*), the identification of a distinct entrance may provide an avenue to design specific inhibitors that target bacterial ATP synthases but not those of the mammalian host.

**Fig. 4.**
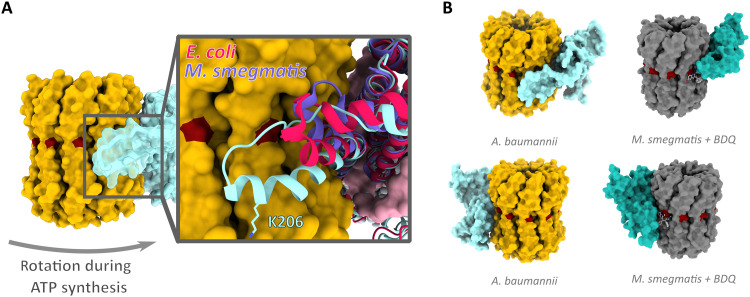
*Acinetobacter*-specific a-subunit extension at the proton exit channel. (**A**) Surface representation of c-ring (yellow) and a-subunit (translucent cyan) viewed from within the membrane plane. Inset: Structural alignment of a-subunits from *A. baumannii* and *E. coli* (PDB: 6OQR) (*39*) and *M. smegmatis* (PDB: 7JGA) (*16*) indicates extent of a-subunit loop insertion. K206 shown in stick representation. (**B**) Comparison of a/c_10_ interface in *A. baumannii* (yellow and light blue) and in *M. smegmatis* [dark gray and turquoise, PDB: 7JGA (*16*)] showing the binding site of BDQ in *M. smegmatis* (light gray ball and sticks); proton-carrying residues are shown in dark red. Bottom panels are rotated by 180° around the membrane normal.

The *A. baumannii* a-subunit contains an additional loop extension between aH4 and aH5 (Fig. 4A). The loop extension is formed by predominantly hydrophobic residues plus a charged lysine residue (^200^PSNPVAKALLIP^211^) and is conserved in the *Acinetobacter* genus and in the Moraxellaceae family but is fully or partially absent and least conserved in other ATP synthases (fig. S5A). A structural alignment of a-subunits from several bacterial ATP synthases (Fig. 4A) reveals that the *A. baumannii* loop is substantially longer than in other bacteria and may even reach the periplasmic membrane edge (fig. S6). In cells, such proximity may provide privileged access to small molecules and biologics, which is absent in other species. In addition, lysine 206 appears to stretch toward the bilayer leaflet, suggesting that it might even interact with phospholipid headgroups (Fig. 4A and fig. S6).

### ATP synthase of bacterial pathogens as drug target

The success of BDQ in treating tuberculosis has ignited interest in targeting ATP synthases in ESKAPE pathogens. Unlike most ATP synthase inhibitors (*18*), BDQ binds with high specificity to mycobacterial ATP synthases. In structures of BDQ bound to the *Mycobacterium smegmatis* ATP synthase, the highest-affinity binding sites were found at the interface between c_10_ and the a-subunit (Fig. 4B) (*16*). Furthermore, the broad-spectrum antimalarial antibiotic mefloquine and the new antibiotic lead tomatidine (*19*) are suggested to act in a similar manner, targeting the a/c-ring interface (*20*).

As the a-subunit loop insertion is conserved within the *Acinetobacter* family but is absent or structurally diverse in other bacteria and the mitochondrial ATP synthase, the unique a/c_10_ interface in *A. baumannii* represents a prime target for the development of highly specific inhibitors. BDQ belongs to a class of small molecules known as diarylquinolines (DARQs). A targeted screen of ~700 DARQs yielded compounds that specifically inhibited the ATP synthase from *Staphylococcus aureus* and *Streptococcus pneumoniae* but showed minimal activity toward *M. tuberculosis* (*21*). These data demonstrate that inhibitors that bind at the c-ring or the a/c_10_ interface can be optimized to target specific ATP synthases beyond *Mycobacteria,* and future structure-based drug design may facilitate the development of compounds targeting this interface in ESKAPE pathogens (*1*). The structure of the *A. baumannii* ATP synthase reveals novel and unique features of a bacterial ATP synthase and will inform future efforts to develop much needed antibiotics to treat MDR *Acinetobacter*.

## MATERIALS AND METHODS

### Chemicals, enzymes, and strains

If not stated otherwise, all chemicals were purchased from Sigma-Aldrich and Life Technologies/Thermo Fisher Scientific.

### Bacterial strains and growth conditions

*A. baumannii* American Type Culture Collection 17978 was grown in tryptone soy broth, Terrific broth, or Lysogeny broth (LB) agar plates at 37°C. *E. coli* Top10 was grown in LB at 37°C with the addition of agar as required. Media were supplemented with antibiotics tetracycline (15 μg/ml) or kanamycin (50 μg/ml) as required.

### Molecular cloning

*A. baumannii* genomic DNA isolation was performed using the PureLink Genomic DNA mini kit (Life Technologies). Isolation of plasmid DNA was carried out using the QIAprep spin miniprep kit (QIAGEN). The β-subunit encoding gene of *A. baumannii* ATP synthase (3815454-3814060, GenBank: CP053098.1) was amplified with KOD Hot Start DNA Polymerase (Novagen) as described by the manufacturer with the inclusion of 1 M betaine using the BetaFstrep forward primer 5′-TCTGACTCGAGTAACAGGAGGAATTAACCATGTGGAGCCACCCGCAGTTCGAAAAGAGTAGCGGTCGTATCATTC-3′ and the BetaR reverse primer 5′-CAGATCTGCAGTTAGAGTTTCTCAGCTTTAGCAA-3′. These primers introduce the optimized pBAD 18–base pair ribosome-binding site and then the sequence encoding a StrepII tag at the gene 5′ end of the gene. The amplified gene was inserted into pCR-Blunt II-TOPO and transformed into *E. coli* Top10 and confirmed by colony polymerase chain reaction (PCR) screening with standard Taq polymerase [New England Biolabs (NEB)] with the addition of 2% dimethyl sulfoxide. The correct insert was then digested from purified plasmid using Xho I and Pst I restriction endonucleases according to the manufacturer’s specifications (Roche). The insert was then ligated into digested pBBR-MCS3 (*22*) before transformation into Top10. The confirmed sequence of plasmid pBBR_AbATPbetaSII was then introduced into *A. baumannii* cells via electroporation. DNA sequences were confirmed by sequencing of all polymerase-amplified regions (GATC Biotech).

### Isolation of ATP synthase from *A. baumannii*

Cultures of *A. baumannii* cells containing vector pBBR_AbATPbetaSII were grown in TB media containing tetracycline (15 μg/ml) overnight and harvested by centrifugation at 5000*g*. All subsequent steps were performed at 4°C. Wet cells (5 g) were resuspended in buffer A [50 mM 4-(2-hydroxyethyl)-1-piperazineethanesulfonic acid (Hepes) buffer (pH 6.8), 150 mM KCl, and 5 mM MgCl_2_] supplemented with a spatula tip of deoxyribonuclease I and cOmplete Protease Inhibitor Cocktail (Roche). Cells were lysed by passaging three times through a French pressure cell at 20,000 psi and centrifuged at 10,000*g* for 1 hour. The remaining supernatant was first filtered through 0.2-μm filters and then centrifuged at 200,000*g* for 1 hour to harvest membranes. The supernatant was discarded, and membranes were resuspended and solubilized in buffer A, which was supplemented by 1% (w/v) of *trans*-4-(*trans*-4′-propylcyclohexyl)cyclohexyl-α-d-maltoside (tPCC-α-M) (Glycon, Luckenwalde, Germany) by gentle agitation for 60 min. The unsolubilized membranes were removed by ultracentrifugation (200,000*g* for 30 min). The supernatant was next loaded onto a Strep affinity column (IBA Lifesciences), equilibrated in buffer B [50 mM Hepes (pH 6.8), 100 mM KCl, and 5 mM MgCl_2_ with 0.05% (w/v) tPCC-α-M] and eluted with buffer B containing 2 mM desthiobiotin using an Äkta Pure chromatography system (GE Healthcare). The eluate was loaded onto a MonoQ 5/50 GL column (GE Healthcare), which was previously equilibrated with buffer B. The product was then gradually eluted with buffer B plus 1 M KCl. Fractions containing the ATP synthase were pooled and desalted using a PD-10 desalting column (GE Healthcare). The enriched ATP synthase was next bound to 0.3 ml of buffer B equilibrated Q Sepharose material for 1 hour. Fractions of 50 to 100 μl were eluted with buffer B plus 500 mM KCl. The purified *A. baumannii* ATP synthase sample was next reconstituted into peptidiscs (Peptidisc Biotech) (*23*) using a peptidisc-to-sample ratio of 2:1 (w/v). Last, sample polishing was performed by size exclusion chromatography (Superose 6 Increase 3.2/300, buffer A, without detergent), and fractions containing the enzyme were selected by SDS-polyacrylamide gel electrophoresis (SDS-PAGE) (*24*) and subsequently pooled (fig. S1, A and B).

### Other biochemical methods

ATP hydrolytic activity was assayed as previously described (*25*). ATP hydrolysis activity was induced by the addition of 0.5% (w/v) lauryldimethylamine oxide and trypsin digestion [1:1 (w/w)] at 37°C. Digestion was stopped by the addition of aprotinin [1:5 (w/w)] after 5 min for 2 min on ice. Protein samples were analyzed by SDS-PAGE (*24*). The protein concentration was determined by the bicinchoninic acid assay (Thermo Fisher Scientific/Pierce) or *A*_280nm_ absorbance via NanoDrop (Thermo Fisher Scientific).

### Sample preparation and cryo-EM

Ultrathin carbon support film, 3 nm on lacey carbon grids (Agar Scientific), was plasma-cleaned in a hydrogen environment for 30 s. Four microliters of purified sample (0.05 mg/ml) was pipetted on the grid in a Vitrobot III [Thermo Fisher Scientific/Field Electron and Ion Company (FEI)] in a 100% humidity chamber at 8°C and frozen in liquid ethane. Images were recorded in a Titan Krios G3 microscope operated at 300 kV (Thermo Fisher Scientific/FEI) with electron-optical alignments adjusted with Sherpa (Thermo Fisher Scientific) on a Gatan K3 direct electron detector in electron counting mode at a nominal magnification of ×5000, corresponding to a calibrated pixel size of 0.425 Å in superresolution mode. A total of 11,490 dose-fractionated movies were recorded using EPU software (Thermo Fisher Scientific/FEI) with an electron flux of 1.42 e^−^ pixel^−1^ s^−1^ over 42 fractions corresponding to a total dose of ~60 e^−^/A^2^ in a defocus range of −1.4 to −3 μm.

### Image processing

Image processing was performed using CryoSPARC throughout the complete image processing procedure (*26*). First, beam-induced motion was corrected, and dose-weighted images from movies for initial image processing were generated. Then, contrast transfer function (CTF) parameters for each movie were determined. Next, particle images were automatically picked and extracted with a box size of 450 × 450 pixels. The dataset was cleaned by two-dimensional classification, and several iterations of ab initio reconstruction and heterologous refinement resulted in three final maps (figs. S2 and S3). The maps were refined using nonuniform refinement from 72,317 (state 1), 26,147 (state 2), and 25,428 (state 3) polished particles. Gold standard Fourier shell correlations were calculated from two independently refined datasets to determine the overall resolution of the reconstructions according to the 0.143 Fourier shell correlation criterion (*27*). To improve the reconstruction of the membrane region, focused refinements using a mask excluding the F_1_ subcomplex (α_3_β_3_γε) and a soft-edged mask around the membrane-embedding ab_2_c_10_ subcomplex were applied before local realignment. Local resolution was assessed using the built-in routine, and maps were sharpened in CryoSPARC. All cryo-EM data collection and refinement parameters are available in table S1.

### Model building and refinement

The structure was built into the EM maps in Coot (*28*) using a combination of Phyre2 (*29*) and based on homologous bacterial and chloroplast structures (*10*–*12*). Parts of the a-, b-, c-, γ-, δ- and ε-subunits and the C- and N-termini of the α- and β-subunits were built de novo. The structure was refined by Namdinator (*30*) and Phenix real space refinement (*31*), followed by manual polishing in Coot, using a composite map of the 3.1-Å state 1 map and the 3.7-Å focused map of the F_o_ membrane region. MolProbity (*32*) was used for validation. The depicted state 1 map in all figures and final validations were made using the state 1 map only, without the composite map. Water-accessible regions of the membrane intrinsic F_o_ subcomplex were probed by mapping the interior surface using HOLLOW (*33*). Figures and movies were made with PyMOL, Chimera, or ChimeraX (*34*–*36*). Rotational analysis was performed according to (*12*).

### HOLLOW channel analysis

All proton channel analyses were conducted using HOLLOW 1.3 (*33*) using a grid spacing of 0.5 Å and a surface probe of 0.8 Å. “Dummy waters” were selected manually, and then, all others within 1.8 Å of seed waters were selected to create the surface visualized in the final figures. Analysis of channels was cross-referenced with existing biochemical data and surface charge characteristics of channels where available.

## Supplementary Materials

### This PDF file includes:

Figs. S1 to S6 Table S1 References

## Notes

View/request a protocol for this paper from *Bio-protocol*.
