# Supplementary material for: Structure of ATP synthase from ESKAPE pathogen Acinetobacter baumannii
Source: Sci Adv. 2022 Feb 16;8(7):eabl5966. doi: 10.1126/sciadv.abl5966 (PMC8849298; doi:10.1126/sciadv.abl5966)
Supplement: Supplementary file 1 — Figs. S1 to S6 Table S1 References [file sciadv.abl5966_sm.pdf]

Science Advances
AAAS

Supplementary Materials for

Structure of ATP synthase from ESKAPE pathogen Acinetobacter baumannii

Julius K. Demmer, Ben P. Phillips, O. Lisa Uhrig, Alain Filloux, Luke P. Allsopp, Maike Bublitz, Thomas Meier*

*Corresponding author. Email: t.meier@imperial.ac.uk

Published 16 February 2022, *Sci. Adv.* **8**, eabl5966 (2022)
DOI: 10.1126/sciadv.abl5966

This PDF file includes:

Figs. S1 to S6
Table S1
References

SUPPLEMENTARY FIGURES

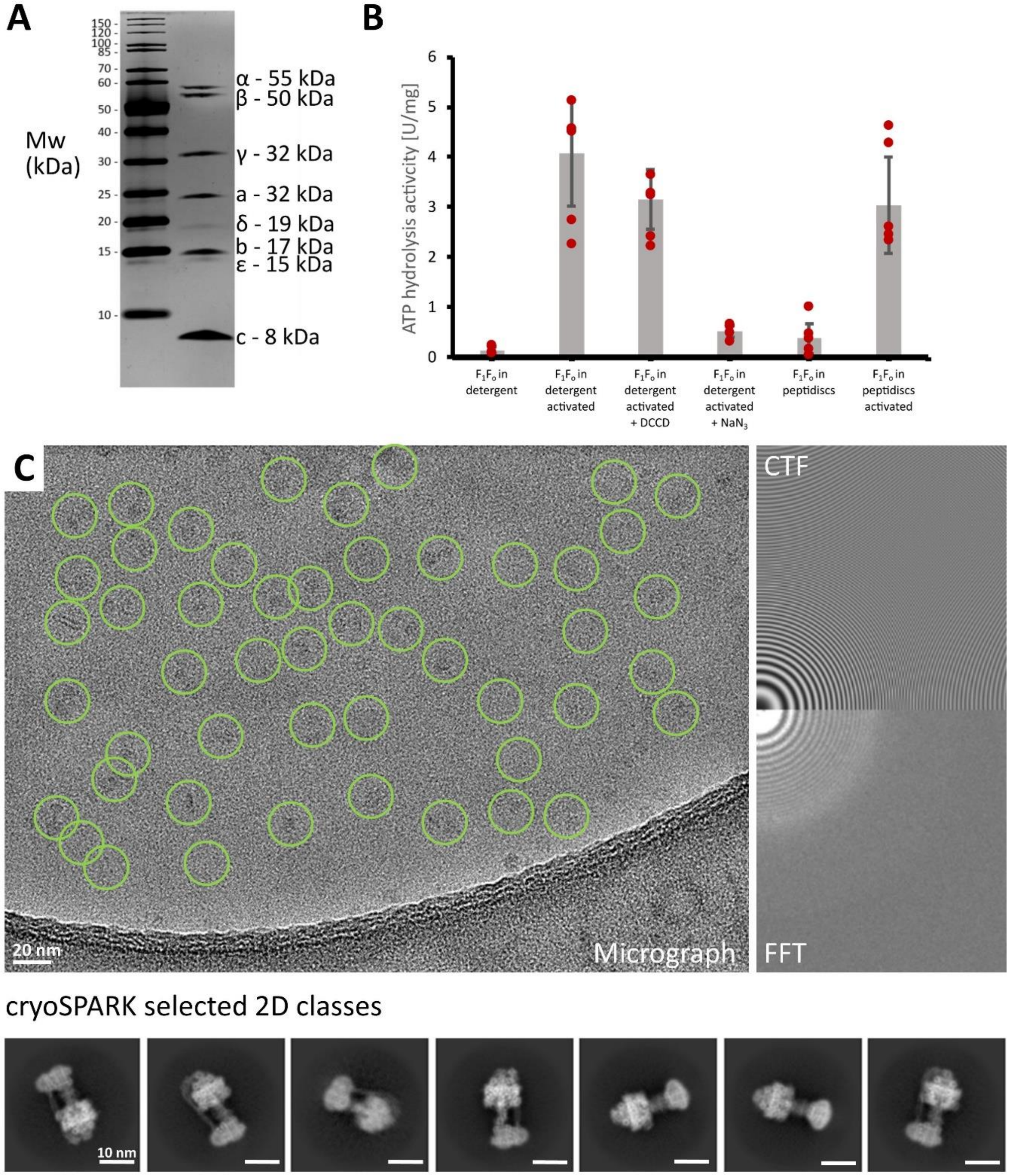

Figure S1 – Purification, biochemical characterisation, and sample quality of the *A. baumannii* ATP synthase. A: Detergent-solubilised ATP synthase on SDS-PAGE (stained with silver). **B:** ATPase hydrolytic activity in the presence of activators (detergent) and inhibitors (DCCD and NaN_3). The ATPase enzyme activity of the purified complex was blocked both in 4-trans-(4-trans-propylcyclohexyl)-cyclohexyl α-maltoside (tPCC-α-M) and peptidiscs but could be restored by addition of lauryl-dimethyl-amine oxide (LDAO) and trypsin to a specific activity of ~4 U/mg in tPCC-α-M and ~3 U/mg in peptidiscs (labelled activated on x-axis). In this activated form, the sample could not be inhibited by the F_o c-ring inhibitor *N*,*N*'-dicyclohexylcarbodiimide (DCCD) but still showed full inhibition by NaN_3, which binds the β-subunit nucleotide binding sites, indicating that F_1 is uncoupled from F_o upon enzyme activation by LDAO/trypsin. **C:** Representative micrograph showing picked particles (green circles), CTF estimation, FFT of raw image and selected 2D classes of *A. baumannii* ATP synthase processed in CryoSPARC version 2.15.

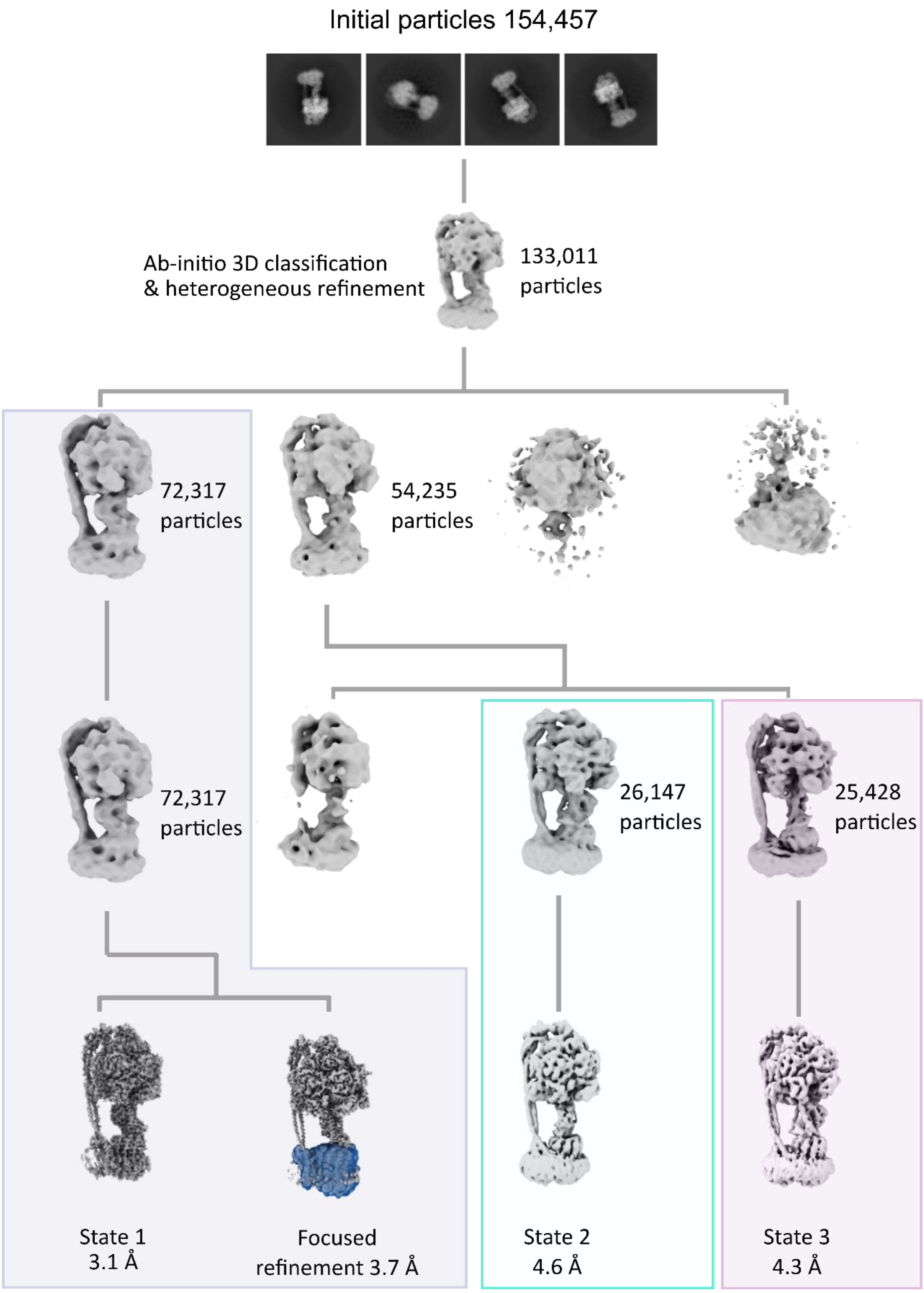

Figure S2 – Flowchart for *cryo*-EM data processing. Particles were subjected to multiple rounds of picking and refinement as indicated. Different rotational states were separated by 3D classification, with each class selected for further processing to high resolution as shown. Once final particles had been selected final rounds of polishing, non-uniform refinement and post-processing resulted in 3 maps with global resolution ranges of 3.1 Å – 4.6 Å. Further focused refinement of the membrane-embedded F_o region in the state 1 map (blue) improved the resolution to 3.7 Å for this region (**Fig. S3C**). All processing was conducted in CryoSPARC 2.15.

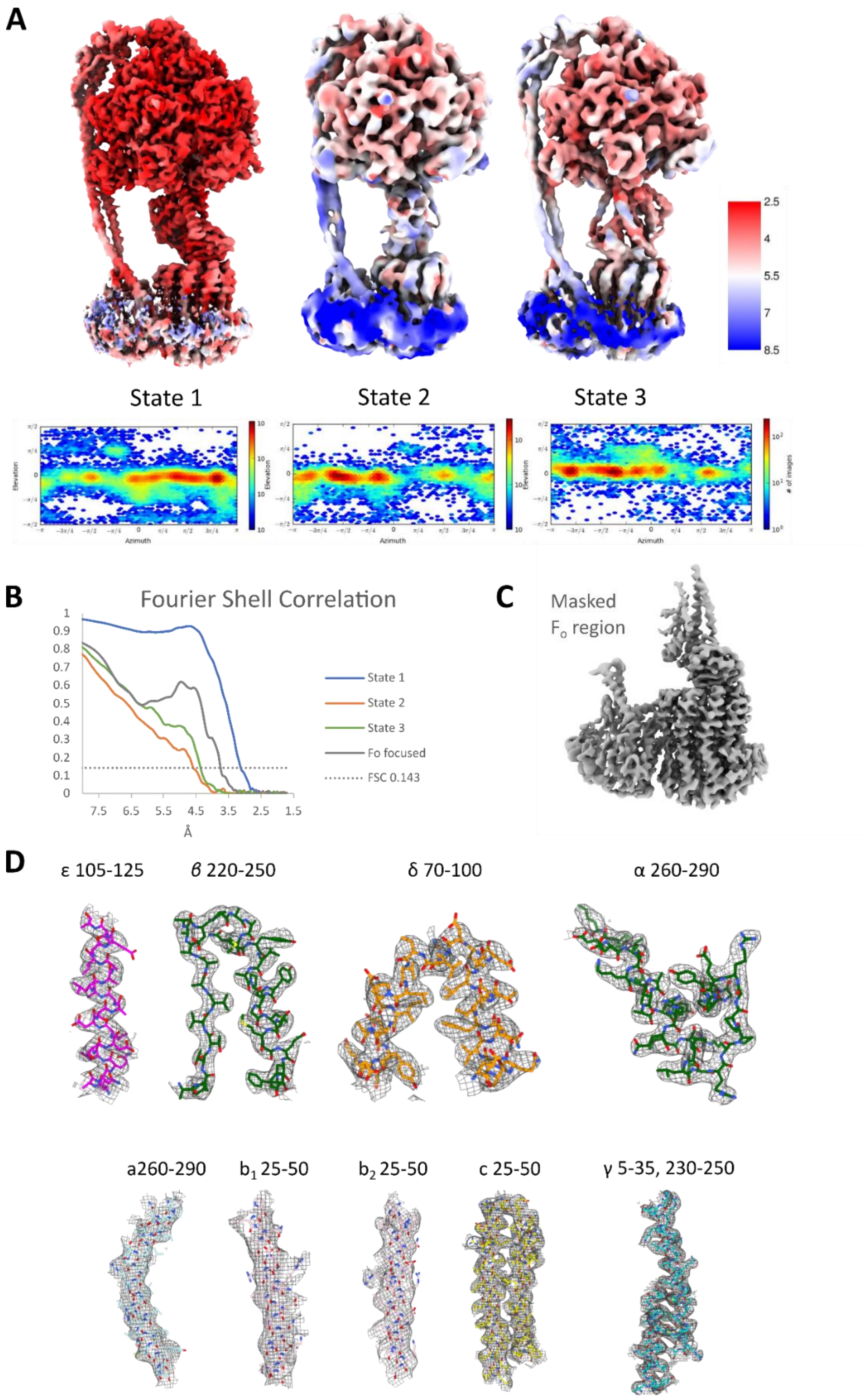

Figure S3 – Quality of EM maps of *A. baumannii* ATP synthase. A: Maps of 3 rotational states of ATP synthase coloured according to local resolution (red = high, blue=low). Below each map is the corresponding Euler angle plot (CryoSPARC version 2.15) indicating angular distribution of views observed in 3D reconstruction. **B**: Fourier Shell Correlation (FSC) curves for 4 EM maps as labelled. **C:** Density map of F_o region following masked refinements to improve local resolution to ~3.7 Å resolution. **D**: Details of map features of state 1 in different subunits with examples of atomic models built in the *cryo*-EM maps (displayed amino acid sequences are indicated).

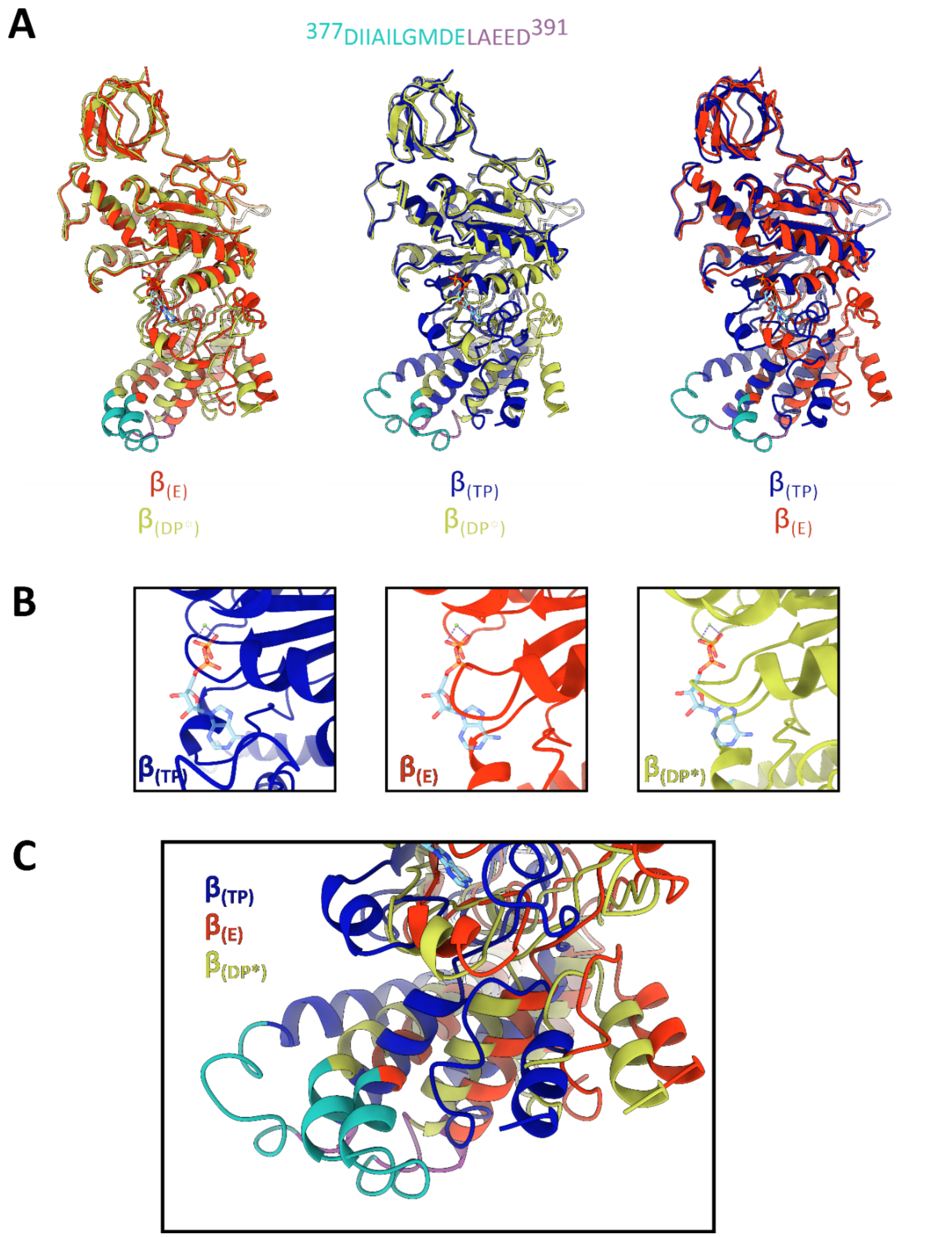

Figure S4 – Conformational changes in the catalytic β subunits of *A. baumannii* ATP synthase. A: Structural alignments of β_{TP} (blue), β_{DP*} (yellow) and β_{E} (red) demonstrate conformational transitions during the ATP synthase catalytic cycle. ADP is shown in stick representation in its binding location in β_{TP} in all 3 alignments for reference only. **B**: Close up of nucleotide binding pockets for all 3 states with ADP as observed in β_{TP} shown in cyan (nitrogen = dark blue, oxygen = red, phosphorous = orange). Steric clashes between the peptide backbone and the ADP in β_{E} and β_{DP*} clearly indicate incompatibility with ATP binding. **C**: Close-up of conformational changes in the ε-proximal helix-turn-helix motif in the β-subunit (^{377}DIIAILGMDE^{386} – shown in cyan) which prevent bidirectional rotation of the central stalk when in the β_{TP} conformation, but not in the β_{E} or β_{DP*} conformations. The state 1 structure (PDB: 7P2Y) was used to generate this figure. All alignments were performed in UCSF ChimeraX.

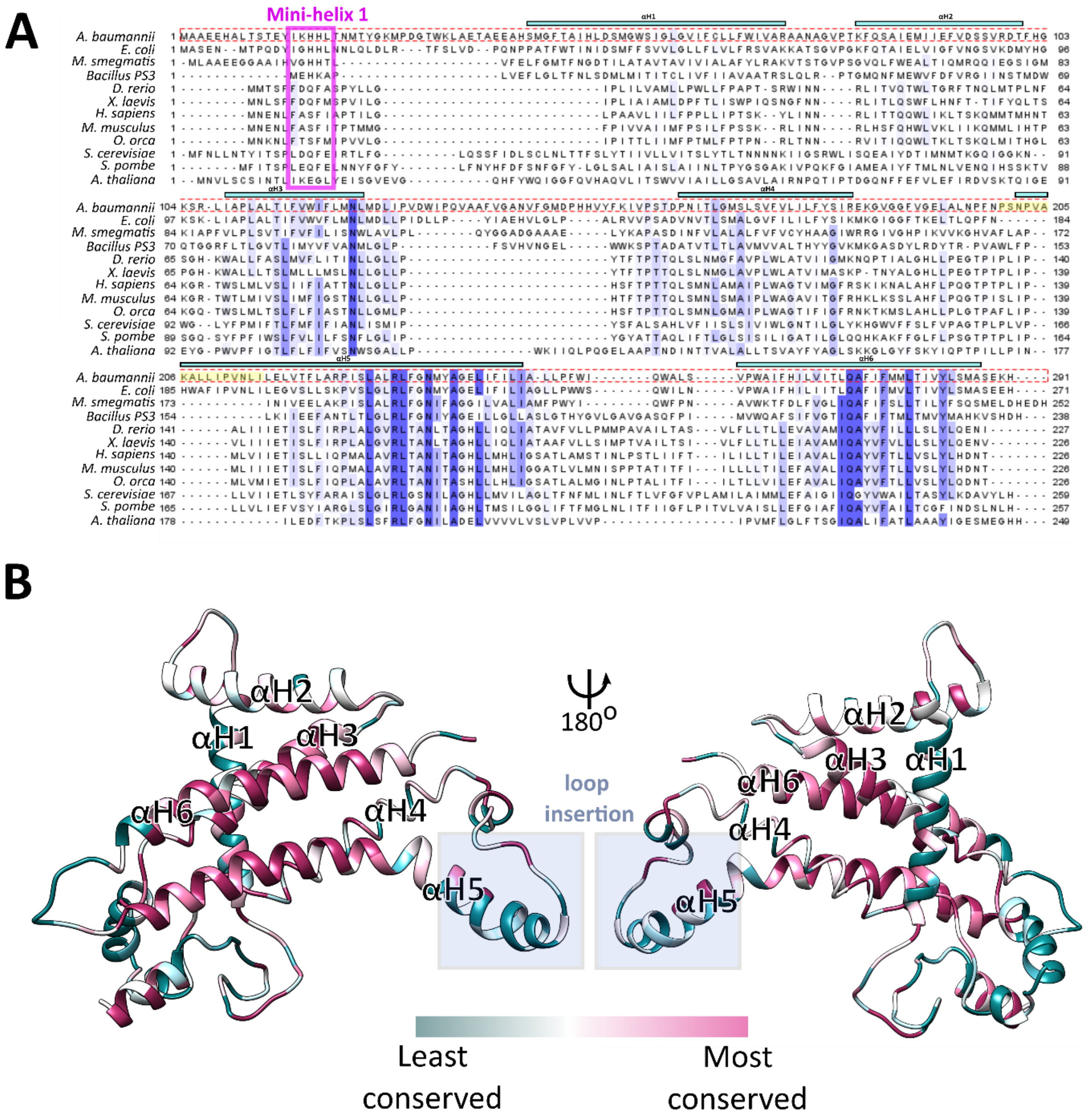

Figure S5 – Amino acid alignment and residue conservation analysis of subunit a of the *A. baumannii* ATP synthase. A: Multiple sequence alignment (produced using ClustalX2 and Jalview) of the a-subunit of the ATP synthase from model organisms with residues coloured by percentage identity across species (white = non conserved, blue = conserved, red dashed box = *A. baumannii* sequence). Purple box indicates residues coloured purple in **Figure 2B** which form mini-helix 1 in *A. baumannii* and *B. taurus.* The locations of the a-subunit helices (aH1 to aH6) are indicated above the alignment. Yellow highlight indicates *A. baumannii* loop insertion between aH4 and aH5. **B**: Cartoon representation of the *A. baumannii* a-subunit coloured according to conservation (bottom) from least conserved (turquoise) to most conserved (pink), generated using the CONSURF server (*40*). The core parts of helices aH5 and aH6 are the highest conserved regions in the a-subunit. Blue box indicates location of a-subunit loop insertion.

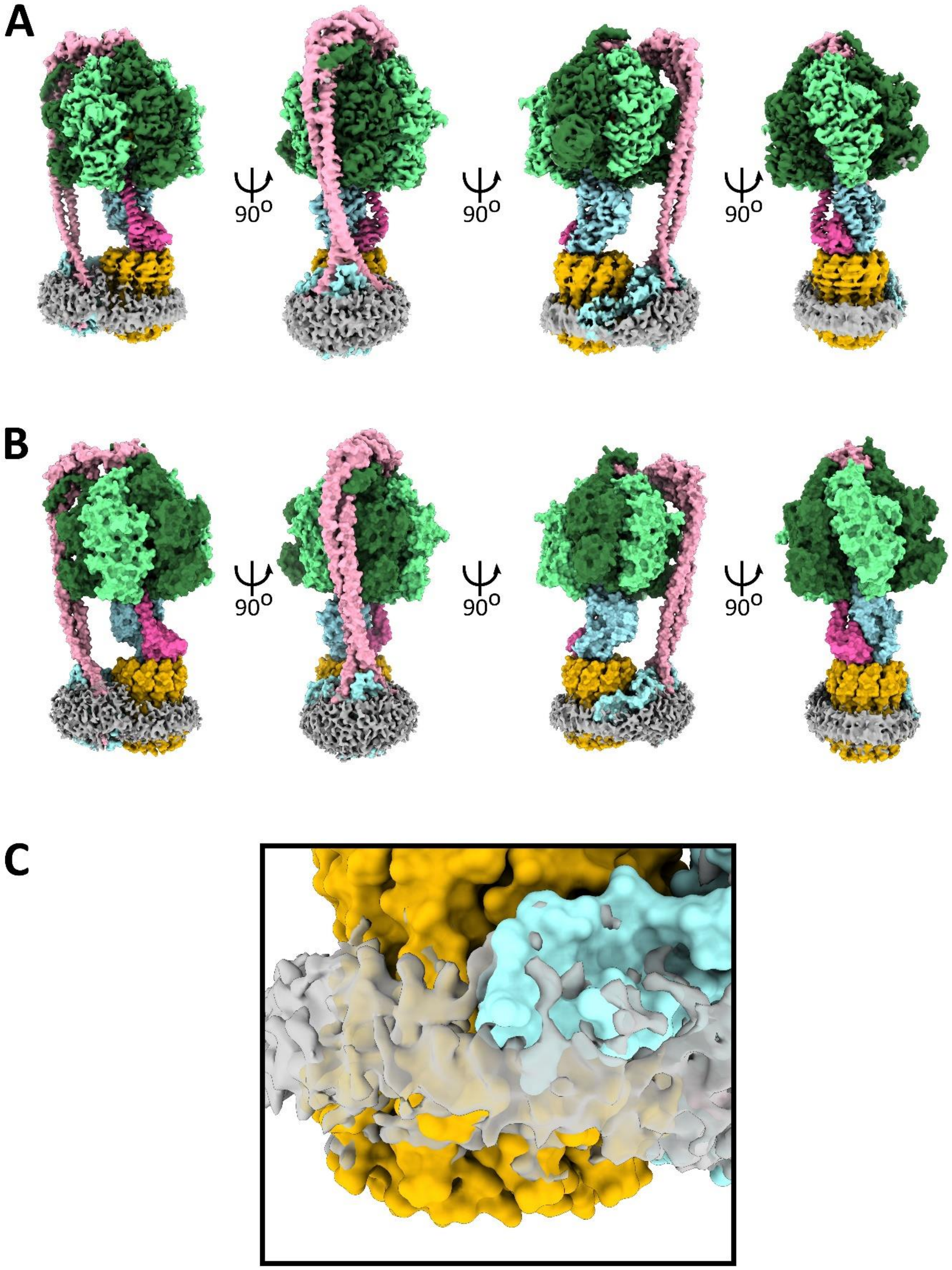

Figure S6 – Peptidisc boundaries of the *A. baumannii* ATP synthase. **A**: Rotated side views of the *cryo*-EM map for *A. baumannii* in peptidisc (state 1), coloured by proximity to main chain in same colour scheme as **Figure 1A**. Map was coloured using UCSF ChimeraX with a 'zone' radius of 5 Å. Note that the peptidisc map is more than 5 Å away from the peptide backbone apart from at the a-subunit loop extension near the periplasmic membrane envelope (view third in from the left). **B**: Surface representation of the *A. baumannii* atomic coordinates file, coloured in correspondence to experimental density in panel (A) with *cryo*-EM map for the peptidisc superimposed (grey density). **C**: Close up view of a surface representation (as seen in panel B, third position) of the a-subunit loop insertion superimposed with the peptidisc *cryo*-EM map.

Table S1 - *Cryo*-EM model building and statistics

	State 1	State 2	State 3
**Data collection**			
Accession numbers	7P2Y<br>EMD-13174	7P3N<br>EMD-13181	7P3W<br>EMD-13186
Electron microscope	Titan Krios G3	Titan Krios G3	Titan Krios G3
Electron detector	Gatan K3	Gatan K3	Gatan K3
Nominal Magnification	85,000	85,000	85,000
Voltage (kV)	300	300	300
Defocus range (µm)	-1.5 - 3	-1.5 - 3	-1.5 - 3
Pixel size (Å)	0.85	0.85	0.85
Total Dose (e^-/ Å²)	60	60	60
Symmetry imposed	C1	C1	C1
Total micrographs	11,490	11,490	11,490
Initial particle images (no.)	349,160	349,160	349,160
Final particle images (no.)	72,317	26,147	25,428
Map resolution (Å)	3.1	4.6	4.3
FSC threshold		0.143	
Map resolution range (Å)	2.6-8.5	3.9-8.5	3.5-8.5
Map sharpening *B* factor (Å^2)	-60	-102	-74
**Image processing**			
Initial model	*De novo*	*De novo*	*De novo*
Model composition			
Protein chains	22	22	22
Non hydrogen atoms	37183	37126	37161
Protein residues	4904	4904	4904
Ligands	3 Mg-ATP<br>1 Mg-ADP<br>1 P_i<br>3 H_2O	3 Mg-ATP<br>1 Mg-ADP<br>1 P_i<br>3 H_2O	3 Mg-ATP<br>1 Mg-ADP<br>1 P_i<br>3 H_2O
R.m.s. deviations			
Bond lengths (Å)	0.004	0.005	0.005
Bond angles (°)	0.716	0.861	0.838
Ramachandran plot			
Favoured (%)	96.2	92.5	93.7
Disallowed (%)	0	0.06	0.02
Validation			
Rotamer outliers (%)	0.08	0.21	0.13
MolProbity Clashscore	10.37	30.55	26.71

REFERENCES AND NOTES

1. M. S. Mulani, E. E. Kamble, S. N. Kumkar, M. S. Tawre, K. R. Pardesi, Emerging strategies to combat ESKAPE pathogens in the era of antimicrobial resistance: A review. *Front. Microbiol.* **10**, 539 (2019).

2. E. M. Duran-Manuel, C. Cruz-Cruz, G. Ibanez-Cervantes, J. C. Bravata-Alcantara, O. Sosa-Hernandez, L. Delgado-Balbuena, G. Leon-Garcia, I. A. Cortes-Ortiz, M. A. Cureno-Diaz, G. Castro-Escarpulli, J. M. Velez-Resendiz, J. M. Bello-Lopez, Clonal dispersion of *Acinetobacter baumannii* in an intensive care unit designed to patients COVID-19. *J. Infect. Dev. Ctries.* **15**, 58–68 (2021).

3. E. Tacconelli, E. Carrara, A. Savoldi, S. Harbarth, M. Mendelson, D. L. Monnet, C. Pulcini, G. Kahlmeter, J. Kluytmans, Y. Carmeli, M. Ouellette, K. Outterson, J. Patel, M. Cavaleri, E. M. Cox, C. R. Houchens, M. L. Grayson, P. Hansen, N. Singh, U. Theuretzbacher, N. Magrini; WHO Pathogens Priority List Working Group, Discovery, research, and development of new antibiotics: The WHO priority list of antibiotic-resistant bacteria and tuberculosis. *Lancet Infect. Dis.* **18**, 318–327 (2018).

4. S. Khoshnood, M. Goudarzi, E. Taki, A. Darbandi, E. Kouhsari, M. Heidary, M. Motahar, M. Moradi, H. Bazyar, Bedaquiline: Current status and future perspectives. *J. Glob. Antimicrob. Resist.* **25**, 48–59 (2021).

5. A. Siroy, P. Cosette, D. Seyer, C. Lemaitre-Guillier, D. Vallenet, A. Van Dorsselaer, S. Boyer-Mariotte, T. Jouenne, E. De, Global comparison of the membrane subproteomes between a multidrug-resistant *Acinetobacter baumannii* strain and a reference strain. *J. Proteome Res.* **5**, 3385–3398 (2006).

6. V. Tiwari, J. Vashistt, A. Kapil, R. R. Moganty, Comparative proteomics of inner membrane fraction from carbapenem-resistant *Acinetobacter baumannii* with a reference strain. *PLOS ONE* **7**, e39451 (2012).

7. T. C. Umland, L. W. Schultz, U. MacDonald, J. M. Beanan, R. Olson, T. A. Russo, In vivo–validated essential genes identified in *Acinetobacter baumannii* by using human ascites overlap poorly with essential genes detected on laboratory media. *MBio* **3**, e00113-12 (2012).

8. N. Wang, E. A. Ozer, M. J. Mandel, A. R. Hauser, Genome-wide identification of *Acinetobacter baumannii* genes necessary for persistence in the lung. *MBio* **5**, e01163-14 (2014).

9. M. Sobti, C. Smits, A. S. Wong, R. Ishmukhametov, D. Stock, S. Sandin, A. G. Stewart, Cryo-EM structures of the autoinhibited *E. coli* ATP synthase in three rotational states. *eLife* **5**, e21598 (2016).

10. H. Guo, T. Suzuki, J. L. Rubinstein, Structure of a bacterial ATP synthase. *eLife* **8**, e43128 (2019).

11. M. Sobti, R. Ishmukhametov, J. C. Bouwer, A. Ayer, C. Suarna, N. J. Smith, M. Christie, R. Stocker, T. M. Duncan, A. G. Stewart, Cryo-EM reveals distinct conformations of *E. coli* ATP synthase on exposure to ATP. *eLife* **8**, e43864 (2019).

12. A. Hahn, J. Vonck, D. J. Mills, T. Meier, W. Kühlbrandt, Structure, mechanism, and regulation of the chloroplast ATP synthase. *Science* **360**, eaat4318 (2018).

13. J. P. Abrahams, A. G. W. Leslie, R. Lutter, J. E. Walker, Structure at 2.8 Å resolution of F_1-ATPase from bovine heart mitochondria. *Nature* **370**, 621–628 (1994).

14. P. D. Boyer, The ATP synthase—A splendid molecular machine. *Annu. Rev. Biochem.* **66**, 717–749 (1997).

15. N. B. Shah, T. M. Duncan, Aerobic growth of *Escherichia coli* is reduced, and ATP synthesis is selectively inhibited when five C-terminal residues are deleted from the ϵ subunit of ATP synthase. *J. Biol. Chem.* **290**, 21032–21041 (2015).

16. H. Guo, G. M. Courbon, S. A. Bueler, J. Mai, J. Liu, J. L. Rubinstein, Structure of mycobacterial ATP synthase bound to the tuberculosis drug bedaquiline. *Nature* **589**, 143–147 (2021).

17. C. von Ballmoos, J. Brunner, P. Dimroth, The ion channel of F-ATP synthase is the target of toxic organotin compounds. *Proc. Natl. Acad. Sci. U.S.A.* **101**, 11239–11244 (2004).

18. S. Hong, P. L. Pedersen, ATP synthase and the actions of inhibitors utilized to study its roles in human health, disease, and other scientific areas. *Microbiol. Mol. Biol. Rev.* **72**, 590–641 (2008).

19. M. Lamontagne Boulet, C. Isabelle, I. Guay, E. Brouillette, J. P. Langlois, P. E. Jacques, S. Rodrigue, R. Brzezinski, P. B. Beauregard, K. Bouarab, K. Boyapelly, P. L. Boudreault, E. Marsault, F. Malouin, Tomatidine is a lead antibiotic molecule that targets *Staphylococcus aureus* ATP synthase subunit C. *Antimicrob. Agents Chemother.* **62**, e02197-17 (2018).

20. A. J. Martin-Galiano, B. Gorgojo, C. M. Kunin, A. G. de la Campa, Mefloquine and new related compounds target the F_0 complex of the F_0F_1 H^+-ATPase of *Streptococcus pneumoniae*. *Antimicrob. Agents Chemother.* **46**, 1680–1687 (2002).

21. W. Balemans, L. Vranckx, N. Lounis, O. Pop, J. Guillemont, K. Vergauwen, S. Mol, R. Gilissen, M. Motte, D. Lancois, M. De Bolle, K. Bonroy, H. Lill, K. Andries, D. Bald, A. Koul, Novel antibiotics targeting respiratory ATP synthesis in Gram-positive pathogenic bacteria. *Antimicrob. Agents Chemother.* **56**, 4131–4139 (2012).

22. M. E. Kovach, P. H. Elzer, D. S. Hill, G. T. Robertson, M. A. Farris, R. M. Roop II, K. M. Peterson, Four new derivatives of the broad-host-range cloning vector pBBR1MCS, carrying different antibiotic-resistance cassettes. *Gene* **166**, 175–176 (1995).

23. M. L. Carlson, J. W. Young, Z. Zhao, L. Fabre, D. Jun, J. Li, J. Li, H. S. Dhupar, I. Wason, A. T. Mills, J. T. Beatty, J. S. Klassen, I. Rouiller, F. Duong, The peptidisc, a simple method for stabilizing membrane proteins in detergent-free solution. *eLife* **7**, e34085 (2018).

24. H. Schägger, G. von Jagow, Tricine-sodium dodecyl sulfate-polyacrylamide gel electrophoresis for the separation of proteins in the range from 1 to 100 kDa. *Anal. Biochem.* **166**, 368–379 (1987).

25. G. M. Cook, S. Keis, H. W. Morgan, C. von Ballmoos, U. Matthey, G. Kaim, P. Dimroth, Purification and biochemical characterization of the F_1F_o-ATP synthase from thermoalkaliphilic *Bacillus* sp. strain TA2.A1. *J. Bacteriol.* **185**, 4442–4449 (2003).

26. A. Punjani, J. L. Rubinstein, D. J. Fleet, M. A. Brubaker, cryoSPARC: Algorithms for rapid unsupervised cryo-EM structure determination. *Nat. Methods* **14**, 290–296 (2017).

27. P. B. Rosenthal, R. Henderson, Optimal determination of particle orientation, absolute hand, and contrast loss in single-particle electron cryomicroscopy. *J. Mol. Biol.* **333**, 721–745 (2003).

28. P. Emsley, K. Cowtan, Coot: Model-building tools for molecular graphics. *Acta Crystallogr. D Biol. Crystallogr.* **60**, 2126–2132 (2004).

29. L. A. Kelley, S. Mezulis, C. M. Yates, M. N. Wass, M. J. Sternberg, The Phyre2 web portal for protein modeling, prediction and analysis. *Nat. Protoc.* **10**, 845–858 (2015).

30. R. T. Kidmose, J. Juhl, P. Nissen, T. Boesen, J. L. Karlsen, B. P. Pedersen, Namdinator—Automatic molecular dynamics flexible fitting of structural models into cryo-EM and crystallography experimental maps. *IUCrJ* **6**, 526–531 (2019).

31. P. D. Adams, P. V. Afonine, G. Bunkoczi, V. B. Chen, I. W. Davis, N. Echols, J. J. Headd, L. W. Hung, G. J. Kapral, R. W. Grosse-Kunstleve, A. J. McCoy, N. W. Moriarty, R. Oeffner, R. J. Read, D. C. Richardson, J. S. Richardson, T. C. Terwilliger, P. H. Zwart, PHENIX: A comprehensive Python-based system for macromolecular structure solution. *Acta Crystallogr. D Biol. Crystallogr.* **66**, 213–221 (2010).

32. V. B. Chen, W. B. Arendall, 3rd, J. J. Headd, D. A. Keedy, R. M. Immormino, G. J. Kapral, L. W. Murray, J. S. Richardson, D. C. Richardson, MolProbity: All-atom structure validation for macromolecular crystallography. *Acta Crystallogr. D Biol. Crystallogr.* **66**, 12–21 (2010).

33. B. K. Ho, F. Gruswitz, HOLLOW: Generating accurate representations of channel and interior surfaces in molecular structures. *BMC Struct. Biol.* **8**, 49 (2008).

34. T. D. Goddard, C. C. Huang, E. C. Meng, E. F. Pettersen, G. S. Couch, J. H. Morris, T. E. Ferrin, UCSF ChimeraX: Meeting modern challenges in visualization and analysis. *Protein Sci.* **27**, 14–25 (2018).

35. E. F. Pettersen, T. D. Goddard, C. C. Huang, G. S. Couch, D. M. Greenblatt, E. C. Meng, T. E. Ferrin, UCSF Chimera—A visualization system for exploratory research and analysis. *J. Comput. Chem.* **25**, 1605–1612 (2004).

36. L. Schrödinger, W. DeLano, 2020. PyMOL; www.pymol.org/pymol.

37. T. E. Spikes, M. G. Montgomery, J. E. Walker, Structure of the dimeric ATP synthase from bovine mitochondria. *Proc. Natl. Acad. Sci. U.S.A.* **117**, 23519–23526 (2020).

38. H. Guo, S. A. Bueler, J. L. Rubinstein, Atomic model for the dimeric F_O region of mitochondrial ATP synthase. *Science* **358**, 936–940 (2017).

39. M. Sobti, J. L. Walshe, D. Wu, R. Ishmukhametov, Y. C. Zeng, C. V. Robinson, R. M. Berry, A. G. Stewart, Cryo-EM structures provide insight into how *E. coli* F_1F_o ATP synthase accommodates symmetry mismatch. *Nat. Commun.* **11**, 2615 (2020).

40. H. Ashkenazy, S. Abadi, E. Martz, O. Chay, I. Mayrose, T. Pupko, N. Ben-Tal, ConSurf 2016: An improved methodology to estimate and visualize evolutionary conservation in macromolecules. *Nucleic Acids Res.* **44**, W344-W350 (2016).
